# Characterization of universal features of partially methylated domains across tissues and species

**DOI:** 10.1186/s13072-020-00363-7

**Published:** 2020-10-02

**Authors:** Benjamin E. Decato, Jianghan Qu, Xiaojing Ji, Elvin Wagenblast, Simon R. V. Knott, Gregory J. Hannon, Andrew D. Smith

**Affiliations:** 1 Quantitative and Computational Biology Section, University of Southern California, Childs Way, Los Angeles, California USA; 2 Watson School of Biological Sciences, Howard Hughes Medical Institute, Cold Spring Harbor Laboratory, 1 Bungtown Road, Cold Spring Harbor, New York 11724 USA; 3 Cancer Research UK Cambridge Institute, University of Cambridge, Li Ka Shing Centre, Robinson Way, Cambridge, CB2 0RE UK; 4 Center for Bioinformatics and Functional Genomics, Department of Biomedical Sciences, Cedars-Sinai Medical Center, 8700 Beverly Boulevard, Los Angeles, CA 90048 USA; 5 Princess Margaret Cancer Centre, University Health Network, University of Toronto, Toronto, M5G 1L7 Canada; 6 Department of Molecular Genetics, University of Toronto, Toronto, M5G 1L7 Canada; 7 New York Genome Center, 101 6th Avenue, New York, NY 10013 USA

**Keywords:** Partially methylated domains, DNA methylation, Cancer, Hidden Markov models

## Abstract

**Background:**

Partially methylated domains (PMDs) are a hallmark of epigenomes in reproducible and specific biological contexts, including cancer cells, the placenta, and cultured cell lines. Existing methods for deciding whether PMDs exist in a sample, as well as their identification, are few, often tailored to specific biological questions, and require high coverage samples for accurate identification.

**Results:**

In this study, we outline a set of axioms that take a step towards a functional definition for PMDs, describe an improved method for comparable PMD detection across samples with substantially differing sequencing depths, and refine the decision criteria for whether a sample contains PMDs using a data-driven approach. Applying our method to 267 methylomes from 7 species, we corroborated recent results regarding the general association between replication timing and PMD state, and report identification of several reproducibly “escapee” genes within late-replicating domains that escape the reduced expression and hypomethylation of their immediate genomic neighborhood. We also explored the discordant PMD state of orthologous genes between human and mouse, and observed a directional association of PMD state with gene expression and local gene density.

**Conclusions:**

Our improved method makes low sequencing depth, population-level studies of PMD variation possible and our results further refine the model of PMD formation as one where sequence context and regional epigenomic features both play a role in gradual genome-wide hypomethylation.

## Background

DNA methylation is associated with a variety of gene regulatory functions in mammals, working in concert with histone marks to stably repress transcription. Early studies of DNA methylation in cancer discovered a globally reduced level of methylation, compared to healthy tissue analogues [1, 2]. The development of modern whole-genome bisulfite sequencing (WGBS) allowed for a high-resolution and full-genome view of DNA methylation. One of the most striking features to emerge from the first application of this technique in mammals were partially methylated domains (PMDs), which were observed in a human lung fibroblast cell line but not in embryonic stem cells [3]. Subsequent studies found these broad domains of reduced methylation to be prevalent in cancer methylomes [4, 5], and we can now attribute the aforementioned global hypomethylation observed in early cancer studies to this phenomenon. Further WGBS studies have established PMDs as a universal feature in methylomes of cancers and cultured cells [6–8]. When they exist, PMDs can cover as much as half the genome, with many contiguous domains larger than 1 megabase [8]. In addition to cancers and cultured cells, PMDs have been identified in the placenta at multiple developmental stages and in several species [9–11].

An increasing number of studies have uncovered several genomic and epigenomic features associated with PMDs. They generally reside in gene-sparse genomic locations and coincide with lamina-associated domains and late-replicating regions [5, 7]. Despite this general trend, their locations show some degree of cell-type specificity [9, 12]. Boundaries of PMDs are enriched for genomic regulatory features including promoters and insulators [5], often containing or defined by CTCF sites [13]. CpG island methylation inside PMDs is pronounced in many cancers [14] and subtle but significant in placenta [11], though it is unknown whether this methylation occurs via the same underlying mechanisms as in cancer methylomes. PMDs in a breast cancer cell line largely overlap genomic regions occupied by histone modifications H3K9me3 or H3K27me3 [7], suggesting a link between PMDs and repressive chromatin that has been further validated by association of PMDs with repressive chromatin states identified by ChromHMM [13].

PMDs can be reliably produced by immortalizing human B-lymphocytes with Epstein–Barr virus (EBV; [8]), and can be erased by inducing pluripotency [6]. There is mounting evidence that PMD formation is related to imperfect maintenance of methylation during mitotic replication, and that certain sequence contexts are more susceptible to this imperfect maintenance than others. Gaidatzis et al. [15] was the first to suggest that different sequence contexts inside PMDs could explain some of the variation in methylation levels. Recently, Zhou et al. [16] showed that that widespread hypomethylation of CpGs in the WCGW context occurred in all tissues as a function of mitotic age, and that these CpGs occurred more frequently in regions likely to contain PMDs. This, coupled with new knowledge that hemimethylated states can persist for quite some time in nascent DNA and failure to remethylate these nascent strands before the next round of replication leads to long-term loss of methylation at that CpG site provides a possible model for PMD formation [17].

A major downside of current research on PMDs are the inconsistent methods used to decide whether a sample has PMDs and the ad hoc identification of PMDs in those samples. In almost all studies on PMDs, some degree of manual inspection of the methylation levels in a genome browser or looking for a bimodal distribution of methylation in windows is used to decide whether a sample contains PMDs or not. In this study, we outline a set of axioms useful to more precisely define PMDs, describe an improved method for PMD identification, and impose data-driven cutoffs on the number and size of segments that allow for identification of PMD-containing samples independent of the biological correlates or mechanisms underlying PMD formation.

We used these improved methods to segment 267 methylomes from 7 species and characterized the features of PMDs and their boundaries. We observed lineage-specific enrichment of repeat elements at boundaries and confirmed the well-known association of transcription start and end sites with our improved PMD boundaries. Analysis of the size and depth of conserved PMD regions across contexts supports a model of progressive PMD formation over time. We showed that while PMDs were generally associated with late-replicating regions, there were a set of highly methylated genes we termed “escapees” that evaded their surrounding PMD state and remained highly expressed regardless of their replication time. Lastly, we used syntenic blocks between mouse and human to explore the relationship between sequence and PMD state, and observed that differential PMD state over orthologous genes was directionally linked to differences in gene expression and local gene density, with the PMD occurring over the ortholog with lower expression and local gene density. Taken together, our novel methodology and analysis serve to clarify the contexts in which PMDs exist, improve their identification in experiments with low sequencing depths, and further elucidate the complex relationship between gradual loss of methylation, sequence, and replication timing.

## Results

### Refining the definition of partially methylated domains

Previous studies have frequently pointed out how easily one can see PMDs when visualizing DNA methylation along chromosomes, assuming the scale is appropriate (e.g., several megabases). Unfortunately, to date PMDs have no functional definition. Statistical definitions based on methylation data have, in almost all cases, been tied to procedures for identifying the PMDs. Here we outline a set of necessary properties of PMDs that capture the key observations of previous studies. Importantly, these properties make no reference to genome annotations or properties of the underlying DNA sequence. This is important in avoiding biases in subsequent analysis of PMDs, for example in identifying features that correlate with PMDs.

We claim that the following properties are essential to defining PMDs:

- they have a lower methylation level than the rest of the genome;
- they cover a fraction of the genome that is distinctly larger than the fraction associated with regulatory features (e.g., CGIs, promoters, enhancers, etc.);
- and they are organized as contiguous genomic intervals whose size is distinctly larger than the size of the aforementioned regulatory features.

The reduced methylation level in PMDs has been assumed by all previous studies. The description of PMDs as being “partially” methylated can be misleading, as we will show, but is often the case since the rest of the genome tends to be highly methylated in most mammalian cells. Regarding the total fraction of the genome covered by PMDs, previous studies have reported a range of 20–65% of the genome covered. Despite the differences in methods used to obtain these numbers, the scale is consistent. The organization of PMDs as contiguous intervals having a particular size distribution has also been common to all previous studies. This is apparent in the use of large bins (e.g., 10 kb; [5] and 20 kb; [10]) and in the care taken for these intervals not to be fragmented by features like CpG islands [12].

Existing reports have found substantial overlap between the portions of the genome covered by PMDs in different methylomes [5, 6]. Should it result from a shared underlying cause, it would be desirable for our definition to recapitulate this concordance without forcing it. At the same time, the definition of PMDs should form the basis for determining whether or not a methylome contains PMDs. For a variety of reasons, large intervals of reduced DNA methylation exist in methylomes that, on a global level, do not appear to have PMDs. One example are HOX clusters in embryonic stem cells, each of which are overlapped by tightly co-located regions of hypomethylation. Another example is pericentromeric satellites in human sperm, which appear to have a distinct chromatin structure leading to their hypomethylation during meiosis [18].

Previous studies have applied PMD detection methods to all samples of interest and then used the features of their segmentation to decide whether each sample contained PMDs or not. For example, Lister et al. [6] observed an order of magnitude difference in the total length of segmentation between the cell lines with and without PMDs, and used that to determine which lines had PMDs. Similarly, Berman et al. [5] claimed PMDs are absent from hESCs and primary normal colon based on a low fraction of the genome found to be covered by PMDs using their sliding window approach. While this method of deciding whether PMDs exist or not in a sample is inherently circular, the clear divide between segmentations in PMD-containing and non-PMD-containing methylomes has allowed it to endure as a reliable decision criteria, and is the approach we take in subsequent sections when deciding whether or not a sample contains PMDs.

### Improving PMD identification through dynamic bin size selection

In this manuscript, we present an improved PMD identification method based off of methpipe’s original PMD detection method [19] and then apply it to a large number of samples. This method is a two-state hidden Markov model (HMM) that segments non-overlapping bins of the genome into one of two states: PMD or background. The distributions of methylation levels in these two states are modeled with beta-binomial distributions and the transition and emission parameters of the model are learned through the Baum–Welch algorithm. PMDs are segmented via posterior decoding and boundaries are heuristically sharpened to single-basepair resolution (see Additional files 1, 2 for a description of our improved heuristic). Lastly, false discovery correction filters out small PMDs by segmenting a shuffled version of the methylome and comparing the size distribution of the resulting segments with the unshuffled segments.

A key technical challenge in accurate comparison of PMDs across many samples from different studies is the bias introduced by variable sequencing depth. As sequencing depth decreases, the fraction of bins in the genome with methylation observations decreases, and the accuracy of the methylation estimation inside those bins deteriorates as the observed value discretizes. A natural approach to reducing this bias is to vary the size of these binned regions on a sample-by-sample basis to equalize the amount of information used to do the segmentation. We defined the minimum amount of “sufficient” information in a bin as 40 observations: whether that is 40 observations of a single CpG or single observations of 40 CpGs. 40 observations corresponds to an 80% confidence interval around an observed methylation level of 50% that ranges from 39% to 61%, which allows us to safely interpret the methylation level in a bin as at least being low, medium, or high. Before segmentation occurs, we choose the minimum bin size that yields at least 40 observations in 80% of all bins genome wide. We observed that the majority of human samples in MethBase were sequenced deeply enough to hit the above criteria with the default bin size of 1000 basepairs. However, most PMD-containing mouse samples were sequenced shallowly enough that bin size adjustment affected our segmentation.

**Fig. 1:**
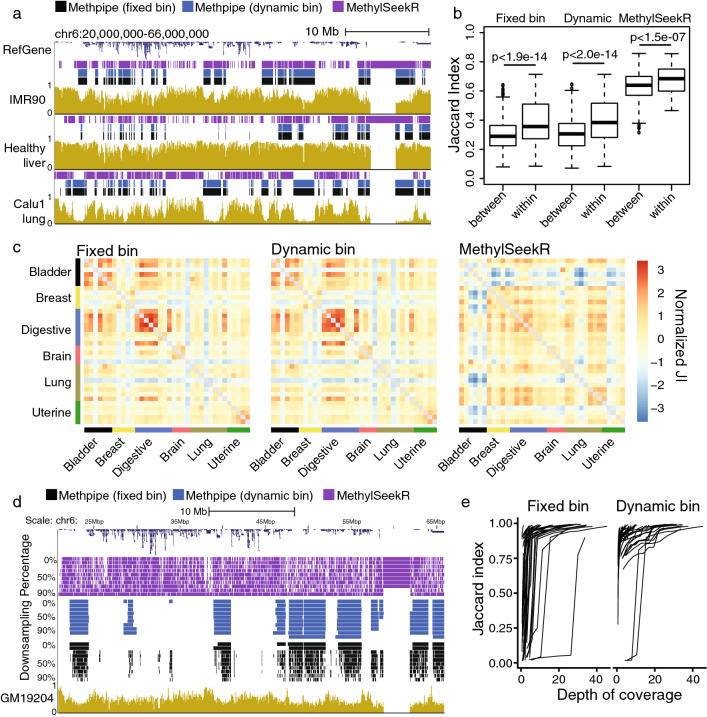
Comparison of PMD identification methods.** a** Comparison of PMD estimates from all three methods on chromosome 6 for three samples with substantial variation in PMD depth.** b** Pairwise Jaccard index distributions comparing within-cancer-type and between-cancer-type for PMD sets identified using each method.** c** Pairwise Jaccard index heatmaps of TCGA cancer sample PMDs identified by each method.** d** Effect of downsampling and low coverage on PMD estimates.** e** Jaccard index of downsampled PMD estimates with their respective full-coverage sample for all human PMD-containing samples

Figure 1a shows the performance of our dynamic bin size method against the fixed bin approach published in methpipe and MethylSeekR’s PMD function [20]. In the first example, all three samples are highly covered and therefore the fixed and dynamic bin estimates are very similar and perform well, with the beta-binomial emission distributions performing flexibly enough to model the very low methylation levels of Calu1 PMDs and subtle methylation differences of the healthy liver sample (which has recently been identified as having PMDs elsewhere [13]). MethylSeekR performs well when methylation levels inside PMDs are near 0.5 and arguably when they are very subtly lower than the background, as in liver, but struggles when PMDs methylation levels are very low, as in the Calu1 lung cancer cell line.

**Fig. 2:**
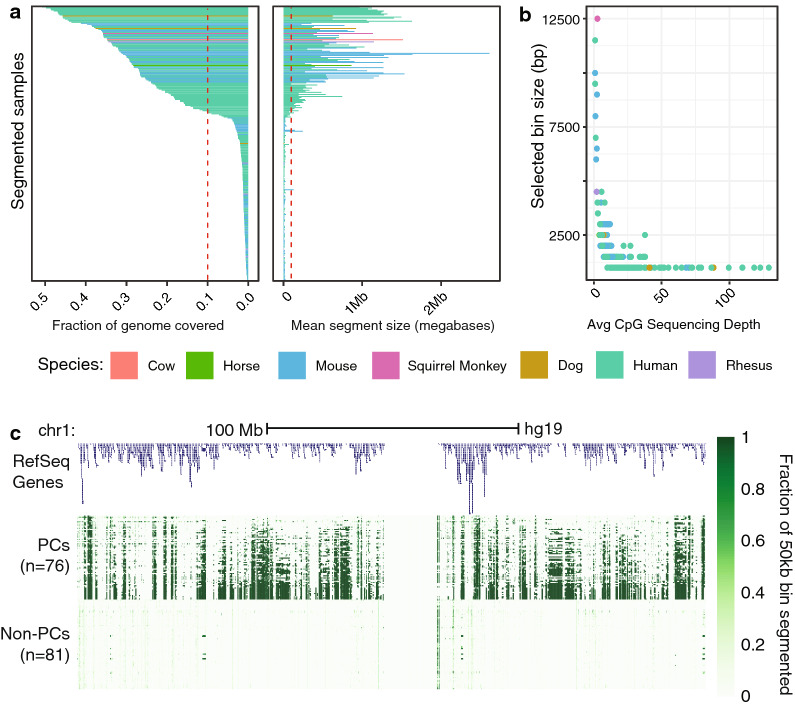
Data-driven decision of PMD state.** a** Fraction of genome covered and mean segment size of segmentation results on all samples studied, with cutoffs drawn to delineate PMD-containing from non-PMD-containing. ** b** Chosen bin sizes and sequencing depth for all samples used in the study. ** c** Genomic locations of segments in PCs vs non-PCs

Because we do not know the underlying process involved in generating PMDs, it is impossible to compare methods against a ground truth, simulated or otherwise. However, we can explore the ability of each method to segregate PMDs identified in a cell-type specific manner. Figure 1b, c shows the normalized Jaccard indices between pairs of tumor samples from The Cancer Genome Atlas (TCGA) for each of the three methods. While MethylSeekR has the highest mean pairwise Jaccard index, the dynamic bin method shows the largest difference in pairwise Jaccard index for pairs of PMD sets coming from the same cancer type vs different cancer types.

Figure 1d shows the performance of each method in extremely low-coverage situations by downsampling. We took a sample from MethBase with very low sequencing depth (50% CpGs covered to 1× depth) and randomly downsampled observations to 90, 80, ... 10% of the original levels. From the manual, MethylSeekR is not recommended for use on samples below 10× sequencing depth and performs poorly as a result. The fixed bin methpipe method performs well at 1× sequencing depth, but suffered from significant erosion of PMD estimates as we downsampled. We show that our method yields consistent PMDs even in the face of extremely low coverage, facilitating future study of PMDs in high-throughput, low-coverage sequencing experiments.

To further show that our variable bin size selection improves the stability of our PMD estimates, we downsampled methylation observations from several samples, segmented the downsampled methylomes using both a fixed 1-kb bin size and dynamically selected bin size, and computed the Jaccard index with the full-coverage PMD segmentation (Fig. 1e). For each sample, we downsampled 9 times: randomly selecting 10%, 20%, ..., 90% of the methylation observations to retain. The resulting plots show that our dynamic bin size selection significantly improves PMD stability in very low-coverage samples by maintaining the segmentation closer to what would be achieved with higher sequencing depth. Interestingly, even with the updated method, we fail to identify accurate PMDs in two samples with the shallowest PMDs (human liver and human H1 mesenchymal cells) until mean CpG sequencing depth exceeds 10×, possibly explaining why PMDs were not observed in these samples until recently.

### Data-driven refinement of the PMD decision criteria

To discern those methylomes that contain PMDs from those that do not, we applied our improved method for PMD detection to a wide range of newly sequenced and public methylomes currently curated in MethBase and TCGA, regardless of whether or not they have been studied in the context of PMDs, and without ascribing a prior on whether they should or should not have PMDs (Additional file 1: Tables S1, S2). Samples were manually annotated as either healthy or cancer, primary or cultured, and with their approximate cell type. To distinguish between PMD-containing (PC) and non-PMD-containing (non-PC) methylomes, we calculated summary statistics based on the properties described in the previous section for each sample. Figure 2a shows the fraction of the genome segmented and the mean size of the segmented regions for all samples analyzed. In all species, there is an inflection point at which both the mean size of segments and total fraction of the genome segmented increases. For the fraction of the genome segmented, the fraction jumps abruptly from roughly 5% (which corresponds well to the fraction of basepairs inside regulatory regions such as gene promoters and CpG islands) to over 10%. At the same time, the mean segment size changes from tens of kilobases to over a hundred. We used these metrics (fraction segmented $>5\%$, mean segment size $>50$kb) as cutoffs to distinguish PC methylomes from non-PC methylomes for the remainder of the study. Our dynamic bin size selection method was critical to selecting these cutoffs in a way that was not influenced by varying sequencing depth. Figure 2b shows the bin sizes selected for segmentation of each PC methylome along with its sequencing depth. Over half of the PMD-containing samples were segmented using a bin size larger than the default of 1000 bp.

After establishing cutoffs, it was clear that there was a substantial difference not only in segment size and frequency but also location in PC samples and non-PC samples. Figure 2c shows a full-chromosome view of the resulting segmentation for all human PC and non-PC methylomes. For each non-overlapping 50-kb region, we colored by the fraction of that region segmented, and observed strong anti-correlation between large segments in PC samples and gene density. Some of the largest segments in non-PC samples came from centromeric regions, which were also segmented in PC samples, and HOX clusters, which were frequently not segmented in PC samples and are likely the result of extended enhancer activity in those regions (Additional file 2: Figure S1). Almost all PC samples fell into the categories of cancer, cultured cell line, or placenta, with some notable exceptions. We corroborated recent results observing PMDs in the human liver [13] and highly divergent but reproducible PMDs in mouse oocytes [10] (Additional file 2: Figure S2).

### Segmentation suggests gradual PMD expansion and conservation of PMD features across species

**Fig. 3:**
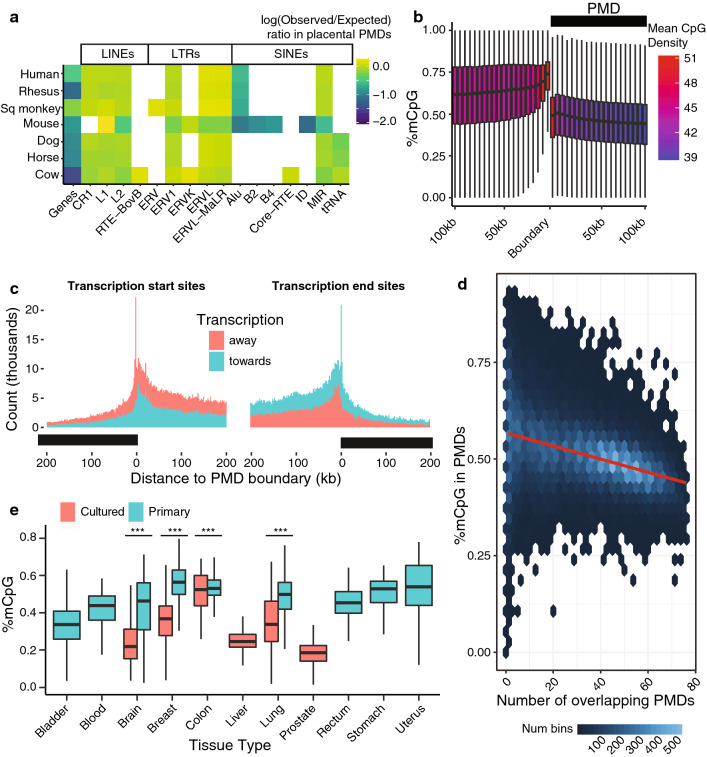
Properties of conserved PMDs.** a** Observed/expected ratios for genes and retrotransposon families inside PMDs. ** b** Metagene plot showing methylation levels and CpG density near boundaries of PMDs. ** c** Histograms showing distance from each RefSeq TSS and TES to its nearest PMD boundary. ** d** PMD depth as a function of 50-kb bin PMD conservation across human samples. ** e** PMD depth boxplots in cultured vs primary cancer samples

Analysis of PMD location overlap with other genomic features revealed significant depletion of genes inside PMDs with a median observed/expected ratio of 0.67 (FDR adjusted $p<0.05$, two-tailed binomial test, Additional file 1: Table S3A), corroborating previous results [5]. Additionally, we observed lineage-specific enrichment of LTR families in primate PMDs, and depletion of nearly all SINE families inside PMDs across species (Figure 3A & Additional file 1: Table S3B/C)). PMDs were observable in non-CpG methylation as well, suggesting differential accessibility of PMD regions by *Dnmt3a* in PC samples vs non-PC samples (Additional file 2: Figure S3).

**Fig. 4:**
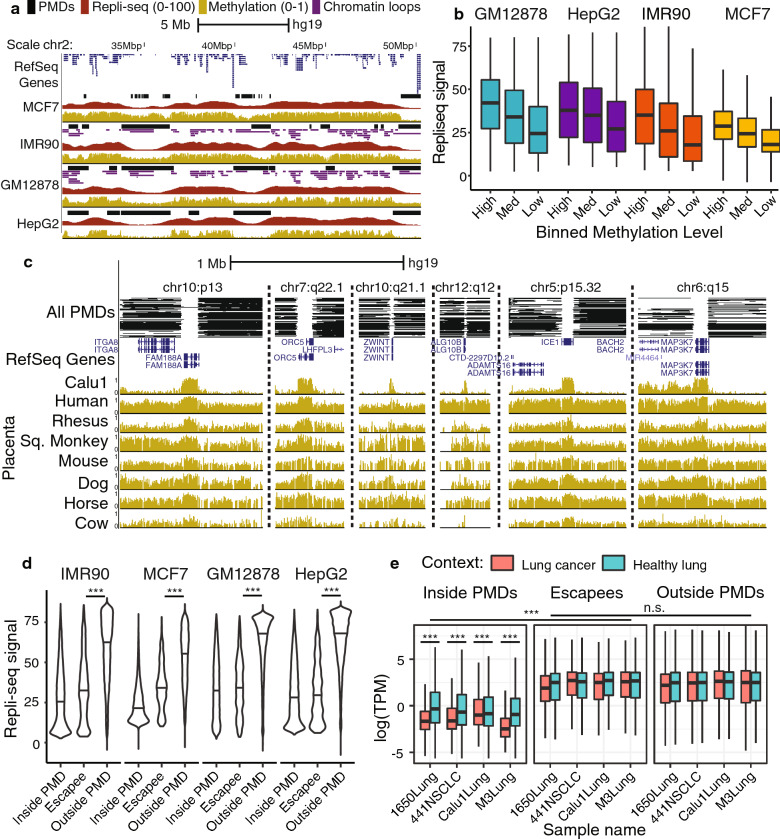
Escapee genes. ** a** Browser plot showing tight association of replication timing and PMD state. ** b** Replication timing for 50-kb bins inside PMDs with high ($>66\%$) medium (33-66%) or low (0–33%) mean methylation level. **c** Stitched browser plot showing 6 most conserved escapee genes in human and their methylation state in other species. **d** Escapee replication timing violin plots. **e** TPM distributions for genes inside PMDs, escapees, and outside of PMDs in PMD-containing samples and healthy analogues

Using all 76 PC human methylomes, we observed that PMD boundaries significantly co-occur with short segments of high CpG density (FDR adjusted $p<0.05$, two-tailed binomial test, Fig.3b , Additional file 1: Table SA), separate domains of high and low CpG density, and that methylation levels inside PMDs increase towards PMD boundaries. This trend is conserved across species (Additional file 2: Figure S4). Elevated CpG density at PMD boundaries was primarily driven by significant enrichment of transcription start and end sites (TSSs and TESs) at PMD boundaries FDR adjusted $p<0.05$, two-tailed binomial test, Additional file 1: Table S4A). TSSs at PMD boundaries displayed a directional affinity for genes transcribing away from the PMD (Fig. 3c). In samples with available CTCF binding data, we observed significant enrichment of CTCF bound sites with PMD boundaries (FDR adjusted $p<0.05$, two-tailed binomial test, Additional file 2: Figure S5, Additional file 1: Table S4B).

We observed only modest enrichment or depletion of repeat families at boundaries relative to the whole genome, but there were substantial family-specific tendencies towards being included or excluded in the PMD if an element occurred at the boundary. We plotted relative enrichment at the boundary using the difference in observed/expected ratios for the 5 kb inside vs 5 kb outside the PMD boundary for each family (Additional file 2: Figure S6). In primate species, Alu elements were preferentially excluded from PMDs, with the magnitude of this exclusion positively correlating with the age of the Alu subfamily. Interestingly, other lineage-specific SINE elements such as the SINEC family specific to dog and equine repeat element (ERE) family specific to horse showed similar exclusion at PMD boundaries.

Next we sought to understand how regional conservation of PMD state across many samples could inform on the progressive appearance and deepening of methylation loss. For every 50-kb bin in the genome that overlapped completely with a PMD in at least one sample, we plotted the number of samples it had a PMD in against its methylation level. We observed that the more conserved a PMD is across samples, the lower its methylation level (Fig. 3d, $\beta = -1.703e$-03, adjusted $r^2=0.1698$, $p<2.2e-16$). This could reflect an ordering, whereby some PMD-covered regions become detectable earlier in their development and have lost more methylation than others over time.

One additional place we explored relative depths of methylation loss was comparing primary cancer samples with their tissue-matched cultured cancer cell line counterparts. We plotted the methylation distribution inside PMDs for cultured vs primary cancer samples, with the assumption that on average, cultured cancer cell lines will have a longer mitotic history than matched-tissue primary tumor samples (Fig. 3e). Cultured cancer samples had lower average methylation levels inside PMDs than their same-cancer primary tissue counterparts. To minimize the possibility of these differences occurring due to stromal composition of primary tumors, we explored the relationship between tumor purity and PMD prevalence in TCGA primary tumor samples and observed no correlation (Additional file 2: Figure S7). This result, while it would benefit from validation in a more purpose-built experiment, points to gradual methylation loss over multiple rounds of mitotic cell division.

### Some genes escape hypomethylation and reduced expression in late-replicating regions

We observed a strong link between late replication timing and PMD state, corroborating previous results [5] (Fig. 4a). PMD boundaries showed significant overlap with chromatin loop boundaries in GM12878 and IMR90 ($p<5.64e-69$ and $p<4.07e-95$; two-tailed binomial test), possibly indicating a significant change in the accessibility of the adjacent regions by Dnmt machinery. In addition, we observed a significant positive relationship between repli-seq signal and methylation level within PMDs in all four samples with matched methylation and repli-seq data (linear regression; one percent change in methylation corresponding to a $\beta =0.52, 0.38, 0.43,$ and 0.42 change in repli-seq signal for IMR90, MCF7, GM12878, and HEPG2, respectively; $p<2.2e-16$ for all four models). This suggests that not only are PMDs associated with late-replication, but the deepest PMD regions are associated with the latest replication times (Fig. 4b). Given the increasingly tight link between late replication and PMD state, we asked whether any genes in predominantly PMD genomic regions escape their hypomethylating and downregulating effects. To explore this, we filtered for all genes in mouse and human whose gene bodies were less than 20% covered by a PMD, and whose 100-kb flanking regions were both at least 80% covered by a PMD. We deemed these genes “escapee” genes and identified 195 unique regions in human and 88 unique regions in mouse that harbored at least one escapee and occurred in at least 2 samples.

Figure 4c shows the top 6 highly conserved escapee genes in human from left to right, as well as the methylation profiles of homologous regions across species. A full list of escapees, their genomic coordinates, and an analogous figure for mouse escapees are available in Additional file 1: Table S5 and Additional file 2: Figure S8. We observed modest conservation of escapee behavior across species, and variability in escapee status even within cancer types (Additional file 2: Figure S9). The top hit, *Fam188a*, was an escapee in 37 of 76 human samples. It is an extremely conserved, ubiquitously expressed protein in mammals [21]. Interestingly, it was only covered by a PMD in one of the 76 samples, a lung adenocarcinoma sample, and has been previously identified as a tumor suppressor of non-small-cell lung cancer [22]. Other top hits coded for equally important proteins, specifically key regulators of apoptosis (*MAP3K7*) and mitotic replication such as a subunit of the origin-replication-complex (*Orc5*) and a protein involved in kinetochore function (*ZWINT*) [23].

Analysis of gene locations revealed that escapee genes exhibit similar replication timing profiles to genes inside PMDs, and significantly later replication timing than other genes outside PMDs (Fig. 4d; Wilcoxon rank-sum test; $p<2.2e-16$ for all four samples). To explore whether escapees also evaded PMD-associated expression reduction, we explored the expression profiles of four lung cancer cell lines and compared them to healthy lung samples. All cultured tissues displayed significantly lower transcript-per-million (TPM) distributions for genes inside PMDs than the same genes in the healthy counterpart (one-sided Wilcoxon rank-sum test; $p<2.2e-16$ for each tissue). In all four cultured tissues, escapee genes exhibited significantly higher TPM than genes inside PMDs (Wilcoxon rank-sum test; $p<2.2e-16$) and similar TPM distribution to genes outside of PMDs (Wilcoxon rank-sum test; $p<0.1027$). This implies these specific genes escape both the partial methylation of their surrounding genomic region and the accompanying reduction in expression of their neighbors (Fig. 4e).

### Discordant PMD state across species correlates with expression and gene density differences

**Fig. 5:**
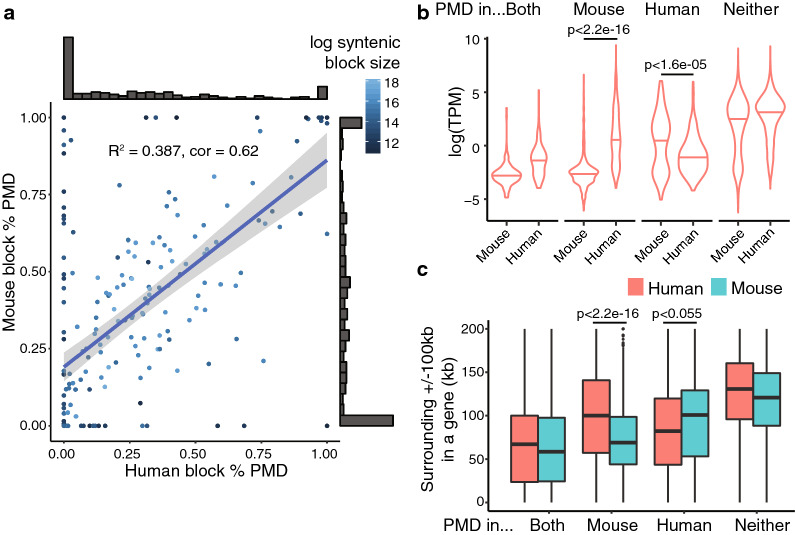
Sequence evolution and PMD state. **a** Syntenic block PMD conservation between mouse and human breast cancer. **b** TPM distributions for orthologous genes between mouse and human binned by PMD state in each species. **c** Comparison of local gene density for orthologous genes between mouse and human by PMD state

Several studies have explored the relationship between large-scale hypomethylation and DNA sequence [15, 16]. We sought to explore the extent to which regions with homologous sequence across species shared their PMD state, and in what cases the PMD state was discordant. To do this, we obtained large (50kb or larger) syntenic blocks between human and mouse based on the human–mouse pairwise alignment first reported by [24]. For each of these blocks, we plotted the percentage of the block covered by breast cancer cell line PMDs in human (HCC1954, [7]) vs mouse (4T1, new samples) in Fig. 5A. The marginal distributions show that most blocks are either entirely covered or entirely uncovered by a PMD in both human and mouse. We observed a significant ($p<2.2e-16$) and positive correlation ($R^2=0.387$), showing that for most homologous regions between human and mouse, their PMD state is shared. Despite this general trend, many blocks displayed highly discordant PMD state regardless of their homologous sequence, with 11 blocks showing greater than 70% discordance. A full table of syntenic blocks analyzed with their PMD state is available in Additional file 1: Table S6.

Looking specifically at the impact of discordant PMD state on genes, we sought to understand whether differential PMD state led to significant directional differences in gene expression. We identified orthologous genes between human and mouse and binned them into four categories: genes with PMDs overlapping them in both species, only in mouse, only in human, or no PMD overlap in either species. We observed significant directional differences in expression for genes that are differentially covered by PMDs ($p<2.2e-16$ for genes in mouse PMDs but not human, and $p<1.6e-05$ for the genes in human PMDs but not mouse; one-sided Wilcoxon rank-sum tests) (Fig.5b).

Lastly, we sought to understand whether orthologous genes with discordant PMD state had differences in neighborhood gene density, which could indicate past genomic recombination events in one species to a region with different heterochromatin state. To explore this, we calculated the local gene density for each orthologous gene by summing the number of basepairs in its surrounding +/– 100 kb that overlapped with other protein coding genes. We observed directional decreases in gene density: genes overlapped by a mouse PMD but not a human PMD had lower local gene density in mouse than human ($p<2.2e-16$), and vice versa ($p<0.0547$; one-sided Wilcoxon rank-sum tests) (5c).

## Discussion

In this study, we described a set of axioms to help improve the definition of partially methylated domains and an improved method for PMD detection. We used these tools together with a large amount of data to provide data-driven cutoffs for whether a sample contains PMDs, and then explored the properties of PMDs across species and contexts.

While PMD locations were fairly consistent across many of the cell types we studied (i.e., occurring in approximately the same 5–30% of the genome) we did some observe cell-type specificity across different cancer types. The sheer size of PMDs, coupled with our method’s ability to identify them in very low-coverage situations, makes them a large and attractive target for early diagnosis of cancer using cell free DNA. We observed significant hypomethylation of satellite-rich pericentromeric regions and HOX gene clusters in non-PC samples. These pericentromeric regions may prove to be a reliable estimator of replication history, given the large variability in methylation loss there in non-PC cell types. HOX cluster hypomethylation seems unlikely to arise via the same gradual loss processes of methylation as PMDs, and PC samples with PMDs covering HOX gene clusters show markedly different methylation patterns.

We observed an increase of methylation levels in PMDs close to the boundary. This increase could reflect some degree of variability in the precise boundary location at the single cell level, or the absence of a precise boundary in favor of increasingly probable hypomethylation of CpG sites as a result of changes in the local environment.

Despite an increasingly tight association between replication timing and progressive loss of methylation, we identified a set of genes residing in late-replicating domains that reliably escape this methylation loss and associated expression reduction. This observation, coupled with the observation that particular histone marks like H3K36me3 co-locate with CpGs that maintain their high methylation in late-replicating regions [16] are contributing to an increasingly clear picture of the major determinants of genome-wide methylation state. It seems likely that starting from some early state following global epigenetic reprogramming in early development, gradual reduction in methylation levels occurs in late-replicating regions. This gradual reduction could roughly track the history of the cells in question, and culminates with identifiable partially methylated domains in cells with a long history of mitotic division, as in liver, placenta, many cancers, and cultured cell lines. Concomitant with this methylation erosion, the actively maintained methylation of critical genes like escapees are exposed like shells at low tide. Mechanisms have been proposed for the preservation of expression in genes close to the lamina, though whether these mechanisms are responsible for escapee methylation is unclear [25].

We observed striking differences in oocyte PMD locations from the PMDs of any other cell type. Given that oocytes are non-dividing, their PMDs are inconsistent with the gradual, replication-induced methylation loss model of other contexts described above. It is possible that incomplete remethylation following epigenetic reprogramming of primordial germ cells, rather than replication-induced methylation loss, gives rise to oocyte PMDs. Heterochromatin-mediated inaccessibility could explain the long-term lack of remethylation, and the significant differences in PMD locations of oocytes from somatic tissues. Studies to determine the precise timing of changes in heterochromatin state and remethylation of primordial germ cells would add valuable evidence for PMDs as a characteristic signature of heterochromatin state.

The discussion of PMD association with late-replicating domains and sequence is well explored, but disentangling the relative effect of these factors on PMD state has been difficult. While we failed to directly explore the differences in replication timing across species in this manuscript, we observed that for orthologous genes in mouse and human, changes in PMD state were linked to changes in gene expression and local gene density. This result could be related to position-effect variegation [26] on a species level, whereby a genomic rearrangement leading to the juxtaposition of an otherwise active gene close to heterochromatin can lead to its subsequent reduction in expression (and in this case, coverage by a PMD).

One remaining and important unknown related to the model of PMD formation and maintenance is the existence of an “equilibrium” methylation level inside PMDs. Some cultured cell lines have been passaged for a very long time and display intermediate methylation levels in PMDs, while others display near 0% methylation levels in PMDs. The speed at which methylation loss occurs in PMD regions, and where that loss stops, may reflect intrinsic differences in the ability to maintain methylation in these regions between cell types. Non-CpG methylation varies with transient *Dnmt3a* expression [27]. Given that we observed PMD-like variation in non-CpG methylation in human placenta, it seems likely that differences in *Dnmt* expression, as well as relative accessibility of Dnmts to PMD regions, could play a central role in determination of equilibrium PMD depth.

## Conclusion

In this manuscript, we presented work aimed at improving the definition and identification of PMDs, and applied our updated identification method to take a comprehensive look at PMDs across species, cellular contexts, and in conjunction with other genetic and epigenetic data. Our improved method has implications for identification of PMDs in extremely low-coverage and high-throughput contexts, including in rare cell types, circulating tumor DNA, or in case–control settings where the number of samples is much larger than their individual sequencing depths. Over the course of our analysis, we validated the increasingly well-documented link between methylation loss and late-replicating domains, and discovered a set of “escapee” genes that reproducibly buck the trend by remaining highly methylated and expressed despite their position in regions of late replication. To the best of our ability given the available data, we showed that these escapee genes are conserved across species, and that they represent a cross-section of genes for which expression appears critical. Left unanswered are interesting questions about why these critical genes remain in late-replicating regions of the genome, how they remain active, and how their variable escapee status in cancer can be used to understand the effects of differential chromatin state on patient outcomes.

## Methods

### An expanded methylome segmentation trackhub

We produced three trackhubs containing methylation, coverage, and PMD segmentations for all the samples analyzed in this study. One contains all PMD-containing methylomes organized by original study, another contains all non-PMD-containing methylomes organized by original study, and the last contains PMD-containing methylomes lifted to distant reference genomes (human to mouse and all species to human). These are public and can be accessed via the UCSC genome browser by entering http://smithlab.usc.edu/lab/public/decato/Decato-PMDs/hub.txt, http://smithlab.usc.edu/lab/public/decato/Decato-nonPMDs/hub.txt, and http://smithlab.usc.edu/lab/public/decato/Decato-Lifted-PMDs/hub.txt, respectively, into the “My hubs” section.

### Mouse mammary tumor cell lines

The parental mouse mammary tumor cell line 4T1 (ATCC) and the derived clonal cell lines were cultured as described in [28]. Genomic DNA was extracted using the QIAmp DNA Blood Mini Kit (Qiagen). Bisulfite sequencing libraries were generated as previously described [29] In short, genomic DNA was fragmented using the Covaris LE220 sonicator to a target fragment size of 200bp. DNA fragments were repaired and ends blunted and phosphorylated. Then, adenylated fragments were ligated to Illumina-compatible paired-end adapters. Subsequently, DNA fragments were purified using the MinElute PCR Purification Kit (Qiagen). Fragments were then denatured and treated with sodium bisulfite using the EZ DNA Methylation-Gold Kit (Zymo Research). Then, the sample was desulfonated and PCR amplified with High Fidelity Expand Plus (Roche) using paired-end adapter-compatible primers. Illumina sequencing was performed, generating 76-nucleotide paired-end reads.

### Lung cancer cell lines and healthy lung tissue

We generated WGBS and RNA-seq libraries for 4 non-small cell lung cancer cell lines and 2 primary normal lung epithelial samples. The cancer cell lines consist of 3 adenocarcinoma (H1650, H441 and M3) and a squamous cell carcinoma (Calu-1). The two primary lung samples are from small airway epithelial (SAE) and bronchial epithelial (BE), respectively. These primary samples, along with the H1650, H441 and Calu-1 cell lines, were obtained from ATCC. The M3 cell line was isolated from H1650 for its resistance to the drug erlotinib and displayed features suggestive of epithelial-to-mesenchymal transition [30]. Genome wide, the WGBS datasets reach 10-15X coverage and cover 93.8%-95.1% of all CpG sites, with bisulfite conversion rates uniformly above 98%.

### Miscellaneous methods

We downloaded genome annotations for repeats [31], CpG islands [32], and human gene bodies [33] using the UCSC table browser [34]. Additionally, we made use of Ensembl biomart to identify orthologous genes across species [35]. The bedtools software suite was used extensively throughout the manuscript [36]. Public Repli-seq [37], CTCF binding [38], and ChromHMM segmentation data [39] were downloaded from the UCSC table browser. Chromatin loop estimates were downloaded from the public data provided by [40]. Asterisks denoting significance in figures were coded as a single asterisk meaning $p<0.05$, two asterisks meaning $p<0.01$, and three asterisks meaning $p<0.001$. Observed/expected overlaps for PMD boundary regions with genomic features such as CpG islands, transcription start sites, and transcription end sites were performed by computing their observed/expected overlap with the +/– 2.5kb surrounding the boundary.

We merged the intra-species placenta methylomes from [11] to produce high-coverage methylomes of the labyrinthine and junctional placental zones. Mouse methylomes from [11] originating from strains other than C57BL/6J were remapped to their recently completed native reference genomes [41] using WALT [42] before being lifted to mm10. All lifts between genome assemblies were performed using the lift-filter program in Methpipe, which acts as a methylation-aware wrapper for the liftOver tool [43]. No chainfile existed for bosTau8 to hg19, so we lifted it from bosTau8 to hg38 and then from hg38 to hg19.

All RNA-seq libraries were mapped using STAR [44] and processed using HTSeq [45].

## Supplementary information

**Additional file 1: Table S1**. Data used in this manuscript with metadata information and source.** Table S2**. Segmentation statistics for all samples studied in this manuscript.** Table S3**A. Gene observed/expected ratios, all human samples. B Gene observed/expected ratios, placental samples by species. C: Retrotransposon family observed/expected ratios by species.** Table S4**A: PMD boundary enrichment statistics.**Table S5**. List of escapee genes for mouse and human.** Table S6**. Syntenic blocks between human and mouse with fraction covered by PMD and coordinates in each species. Discordant if greater than 70% difference in PMD state.

**Additional file 2: Figure S1.** (A) HOX gene clusters display abnormally large, near-complete hypomethylated regions in non-PC samples. (B) Hypomethylation in non-PC samples is prevalent near centromeres and appears linked tosatellite repeat density.** Figure S2**. Pairwise Jaccard index of segmented PMDs in mouse PC samples.** Figure S3**. UCSC genome browser plot comparing CpG methylation levels (yellow) and PMD estimates (black) to the fraction of non-CpG cytosines in 50kb bin that display nonzero methylation levels (green andpink).** Figure S4**. Metagene plot of methylation level and CpG density as a function of distance from PMDboundary stratified by species. Figure S5. Histograms of CTCF bound site distances from PMDs. **Figure S6**. Retrotransposons at PMD boundaries are included/excluded in the PMD in a family-specific manner. Difference in observed/expected ratio for each family by species, with a zoom out showing that theyoungest Alu elements are excluded from PMDs more than the oldest Alu elements.** Figure S7**. Scatterplot of TCGA-reported primary tumor purity against number of basepairs segmented into PMDs.** Figure S8**. Top 6 most conserved mouse escapee genes and their methylation state in homologous regionsof human.** Figure S9.** Adjacent escapee genes showing differential escapee state in TCGA bladder cancer samples.

## Data Availability

Mouse 4T1 and human lung cancer cell line methylomes and expression data have been deposited to GEO under GEO accession number GSE152819. Code used to generate figures is available at https://github.com/bdecato/PMD_Paper_Scripts. Updates to the PMD identification program that underlies analysis described in this paper have been integrated into the MethPipe methylation pipeline, available at https://github.com/smithlabcode/methpipe.
