# Supplementary material for: Characterization of universal features of partially methylated domains across tissues and species
Source: Epigenetics Chromatin. 2020 Oct 2;13:39. doi: 10.1186/s13072-020-00363-7 (PMC7532633; doi:10.1186/s13072-020-00363-7)
Supplement: Supplementary file 2 — Additional file 2: Figure S1. (A) HOX gene clusters display abnormally large, near-complete hypomethylated regions in non-PC samples. (B) Hypomethylation in non-PC samples is prevalent near centromeres and appears linked tosatellite repeat density. Figure S2. Pairwise Jaccard index of segmented PMDs in mouse PC samples. Figure S3. UCSC genome browser plot comparing CpG methylation levels (yellow) and PMD estimates (black) to the fraction of non-CpG cytosines in 50kb bin that display nonzero methylation levels (green andpink). Figure S4. Metagene plot of methylation level and CpG density as a function of distance from PMDboundary stratified by species. Figure S5. Histograms of CTCF bound site distances from PMDs. Figure S6. Retrotransposons at PMD boundaries are included/excluded in the PMD in a family-specific manner. Difference in observed/expected ratio for each family by species, with a zoom out showing that theyoungest Alu elements are excluded from PMDs more than the oldest Alu elements. Figure S7. Scatterplot of TCGA-reported primary tumor purity against number of basepairs segmented into PMDs. Figure S8. Top 6 most conserved mouse escapee genes and their methylation state in homologous regionsof human. Figure S9. Adjacent escapee genes showing differential escapee state in TCGA bladder cancer samples. [file 13072_2020_363_MOESM2_ESM.pdf]

1 An improved method for identifying and scoring optimal PMD boundaries

Identification of precise PMD boundaries and quantification of their strength facilitates downstream analysis of strong vs weak boundary correlates and conservation of boundary strength across samples, making it a very desirable metric to have.

A natural candidate for scoring PMD boundaries are posterior transition probabilities, which are directly calculable for each bin in the PMD detection program and report the probability of transitioning into or out of a PMD at each bin. However, these transition posteriors are subject to the arbitrary partitioning of the genome into non-overlapping blocks and therefore can suffer from technical biases related to where the true PMD boundary is in relation to the two bins the posterior is calculated for. A boundary score should be calculated relative to the true boundary position.

We therefore first improved our precise boundary location method. We know that the true boundaries for each PMD lie somewhere inside the two bins where a maximally probable transition took place during posterior decoding. For each such region, there a set of CpG sites $\boldsymbol{C} = \{C_1..C_n\}$ with coverages $N_1..N_n)$ and number of methylated observations $M_1..M_n)$ that are candidate boundaries. The goal is to pick a boundary site C_k from these sites that splits them into a left bin and right bin. Using the weighted methylation level for the left respectively, we aim to select the site k that maximizes a score representing the probability that the site is the true boundary.

Our method uses the learned PMD (foreground) and non-PMD (background) emission distributions (α_f, β_f), (α_b, β_b) and calculates the joint likelihood of each side of the boundary coming from its respective emission distribution. For all values of k, the likelihood was computed using $M_L = \sum_{i=1}^{k} M_i$, $M_R = \sum_{i=k+1}^{n} M_i$, $N_L = \sum_{i=1}^{k} N_i$, $N_R = \sum_{i=k}^{n} N_i$, $p_L = \frac{M_L}{N_L}$ and $p_R = \frac{M_R}{N_R}$:

$$\mathbb{L}(\alpha_f, \beta_f, \alpha_b, \beta_b | p_L, p_R) = \mathbb{P}(p_L, p_R | \alpha_f, \beta_f, \alpha_b, \beta_b) = \mathbb{P}(p_L | \alpha_b, \beta_b)\mathbb{P}(p_R | \alpha_f, \beta_f)$$

$$= \left(\frac{p_L^{\alpha_b - 1}(1 - p_L)^{\beta_b - 1}}{B(\alpha_b, \beta_b)} \right) \left(\frac{p_R^{\alpha_f - 1}(1 - p_R)^{\beta_f - 1}}{B(\alpha_f, \beta_f)} \right)$$

where $B(\alpha, \beta)$ is the Beta function. We considered using the maximized joint likelihood directly as a boundary quality metric, but depending on where the partition is the maximum value of the likelihood may be biased (as in the case of all but one CpGs belonging to the PMD or non-PMD state, respectively). To overcome this, we optimized boundaries first and then for each boundary, take 1 bin on either side and calculate the joint likelihood using that equal weighting.

In order to get a joint likelihood for each boundary that is comparable across the genome, it is important that they are not influenced by coverage. Therefore for each boundary, we calculate the joint likelihoods

assuming a beta distribution rather than beta-binomial, and report the coverage for the bin with less coverage as well as the likelihood as a "certainty score." This helped downstream to know whether we could trust a boundary value, and allowed us to compare the quality of boundaries without worrying about differences in sequencing depths affecting the likelihood.

2 Supplementary Figures

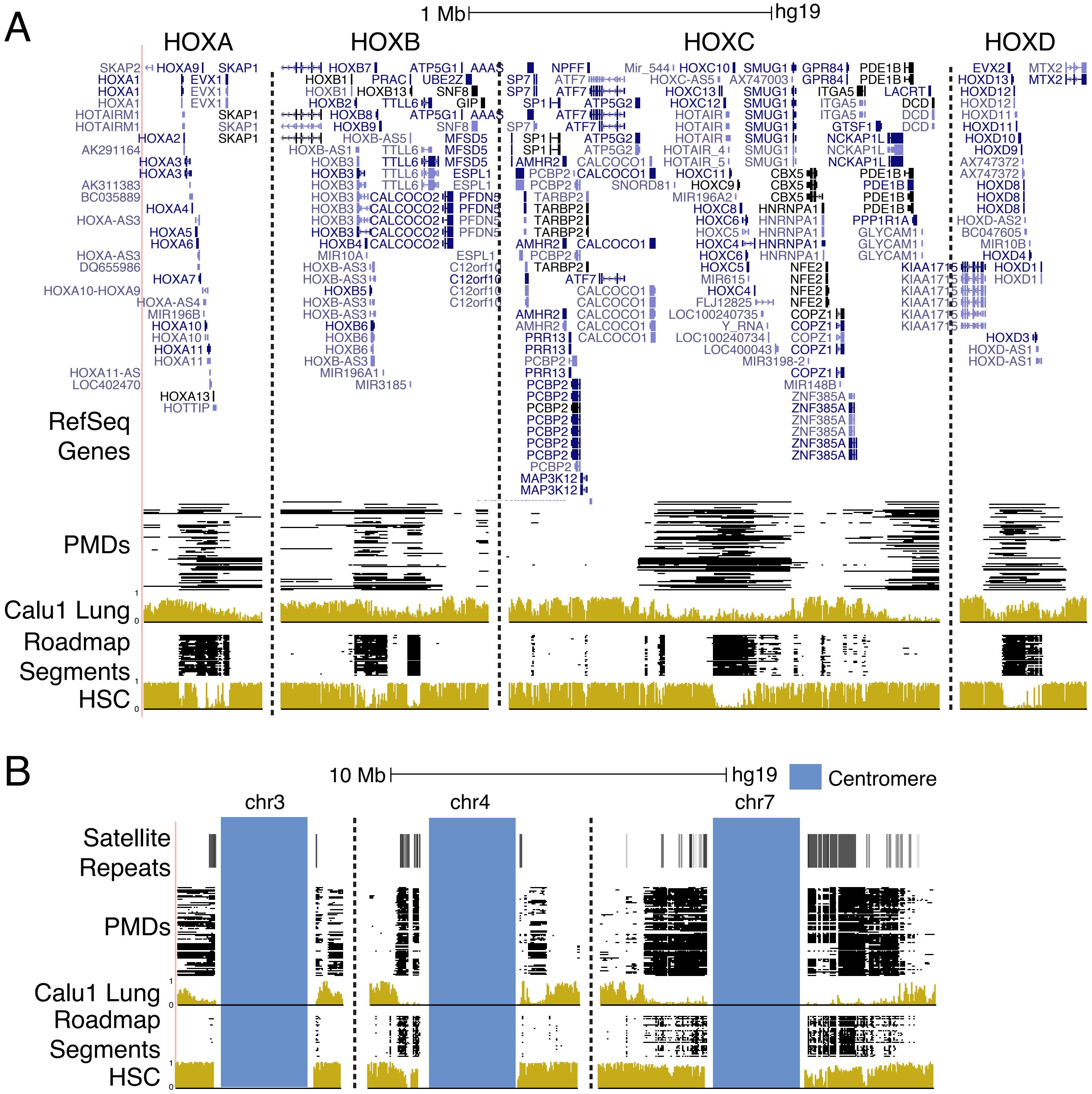

Figure S1. (A) HOX gene clusters display abnormally large, near-complete hypomethylated regions in non-PC samples. (B) Hypomethylation in non-PC samples is prevalent near centromeres and appears linked to satellite repeat density.

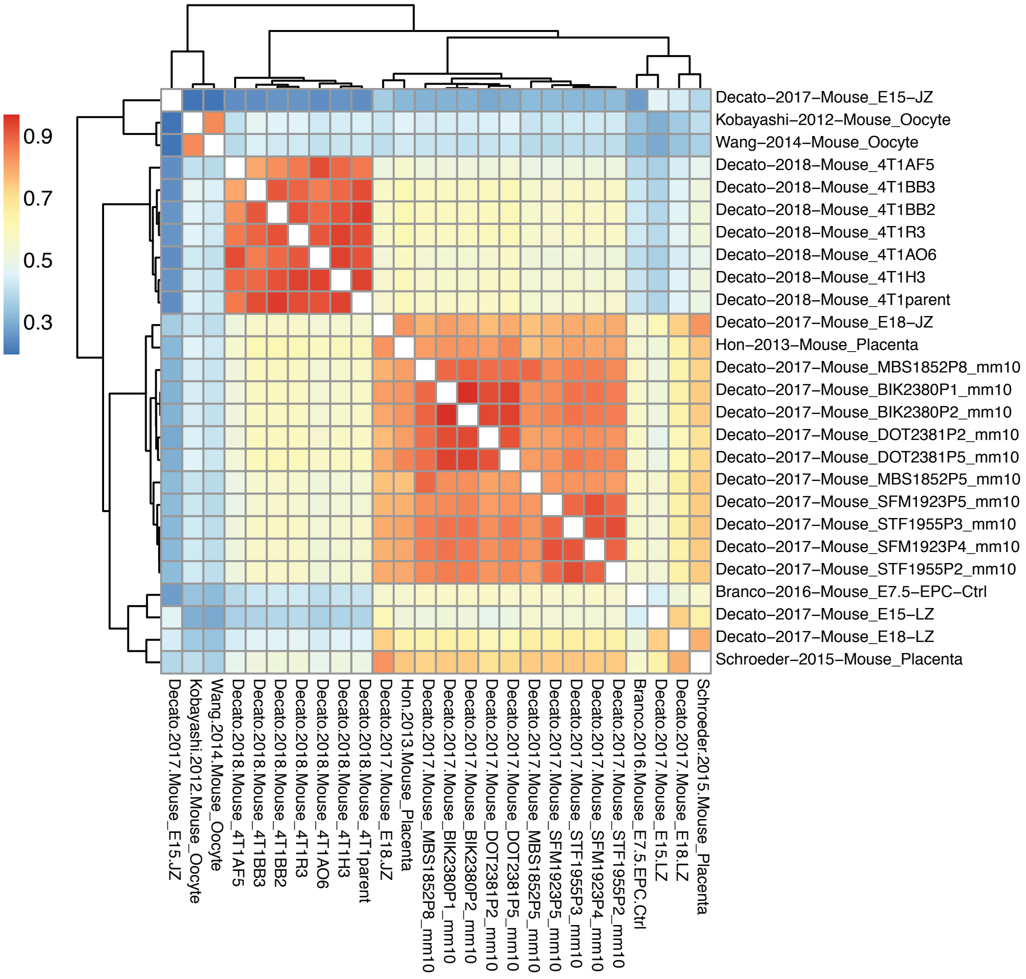

Figure S2. Pairwise Jaccard index of segmented PMDs in mouse PC samples.

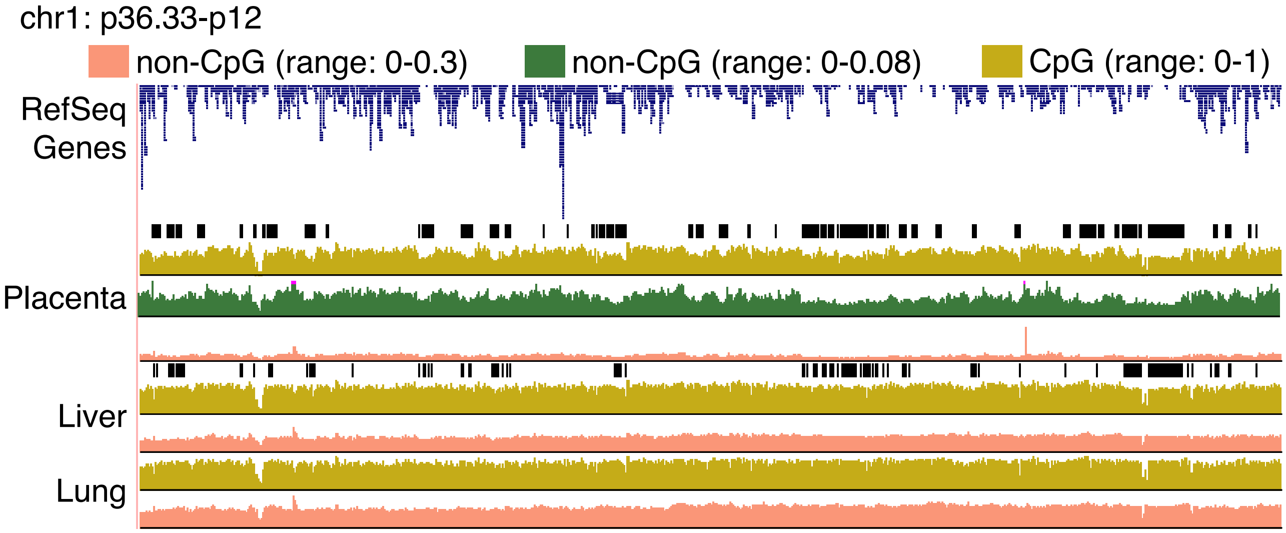

Figure S3. UCSC genome browser plot comparing CpG methylation levels (yellow) and PMD estimates (black) to the fraction of non-CpG cytosines in 50kb bin that display nonzero methylation levels (green and pink).

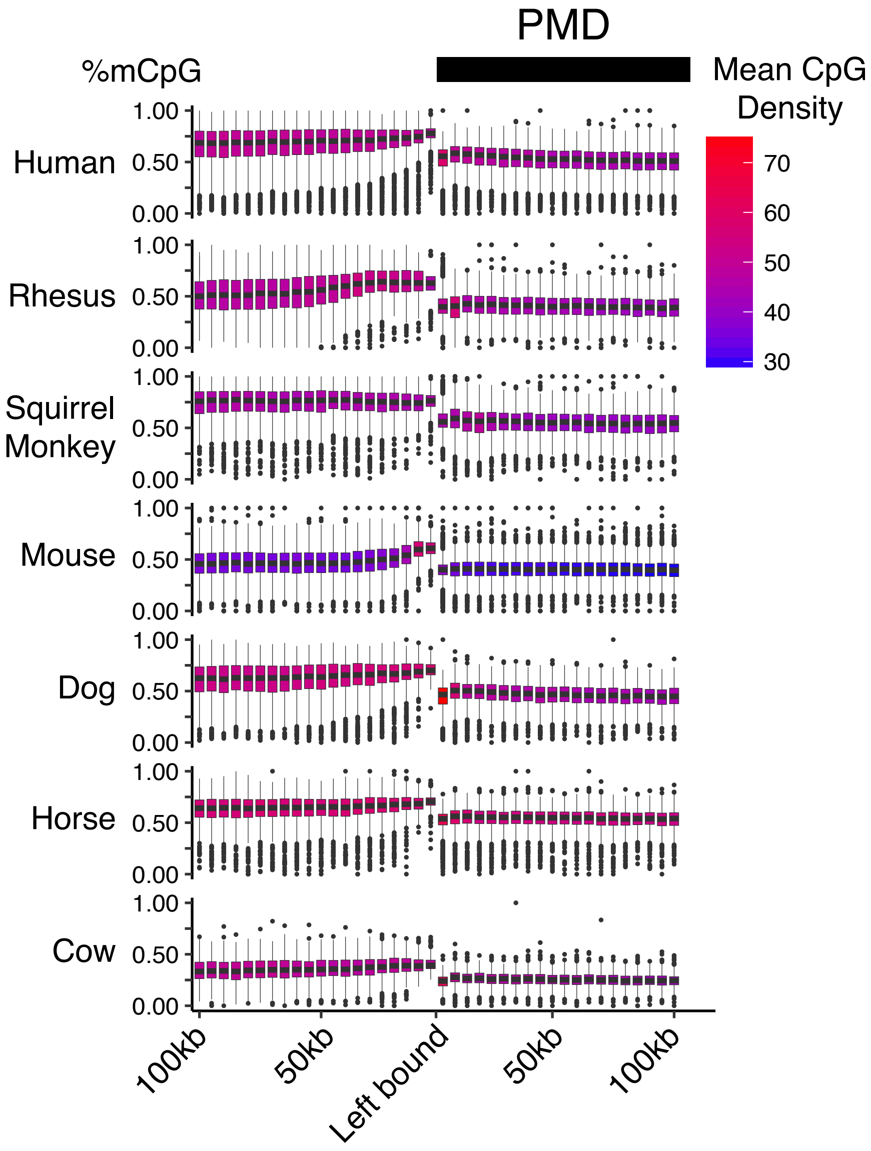

Figure S4. Metagene plot of methylation level and CpG density as a function of distance from PMD boundary stratified by species.

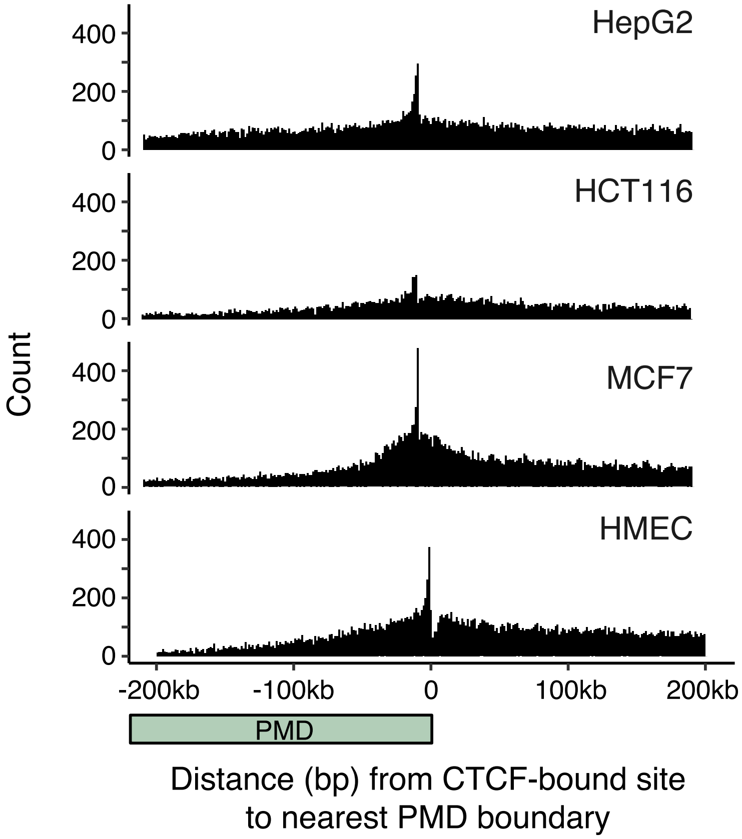

Figure S5. Histograms of CTCF bound site distances from PMDs.

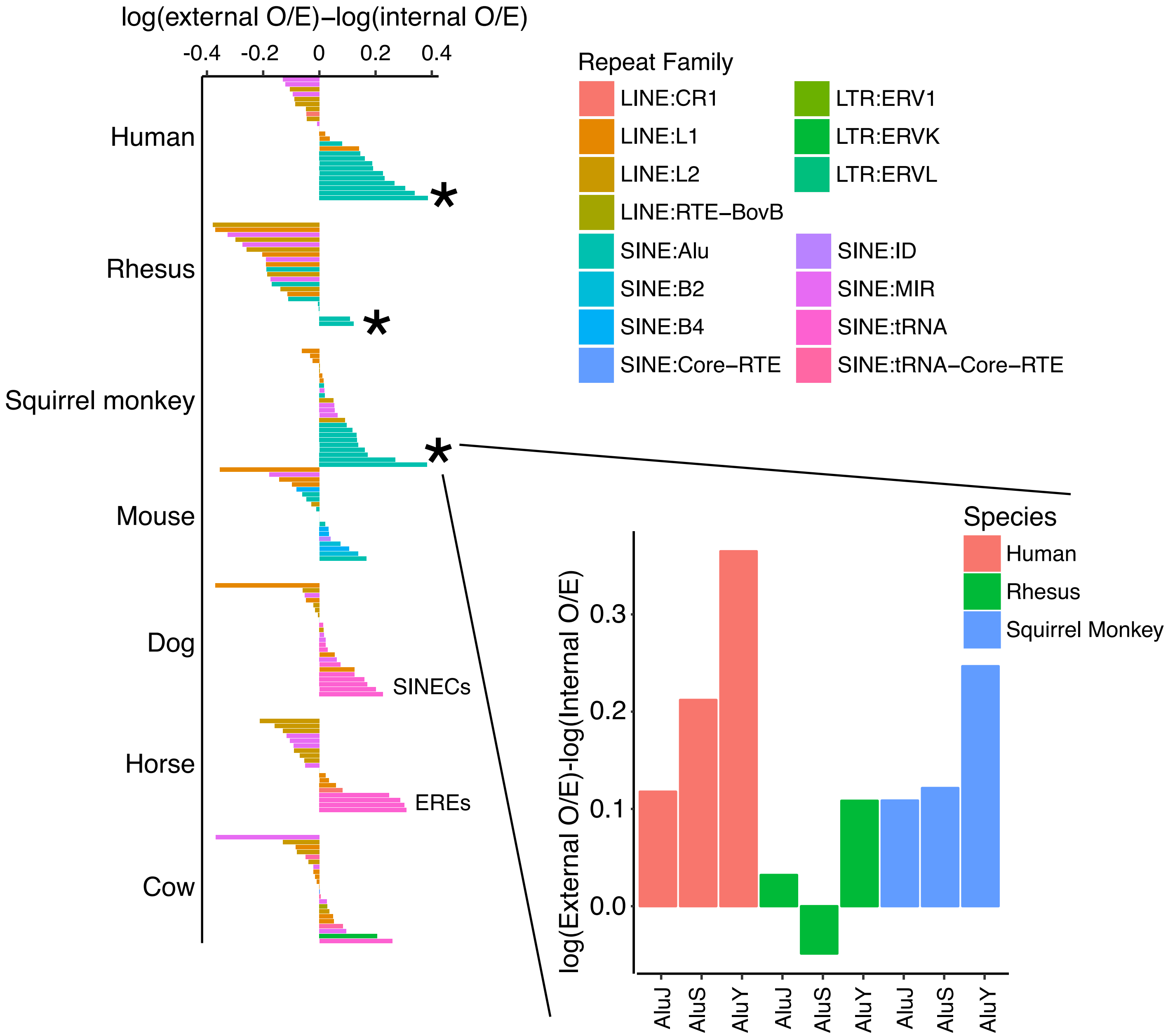

Figure S6. Retrotransposons at PMD boundaries are included/excluded in the PMD in a family-specific manner. Difference in observed/expected ratio for each family by species, with a zoom out showing that the youngest Alu elements are excluded from PMDs more than the oldest Alu elements.

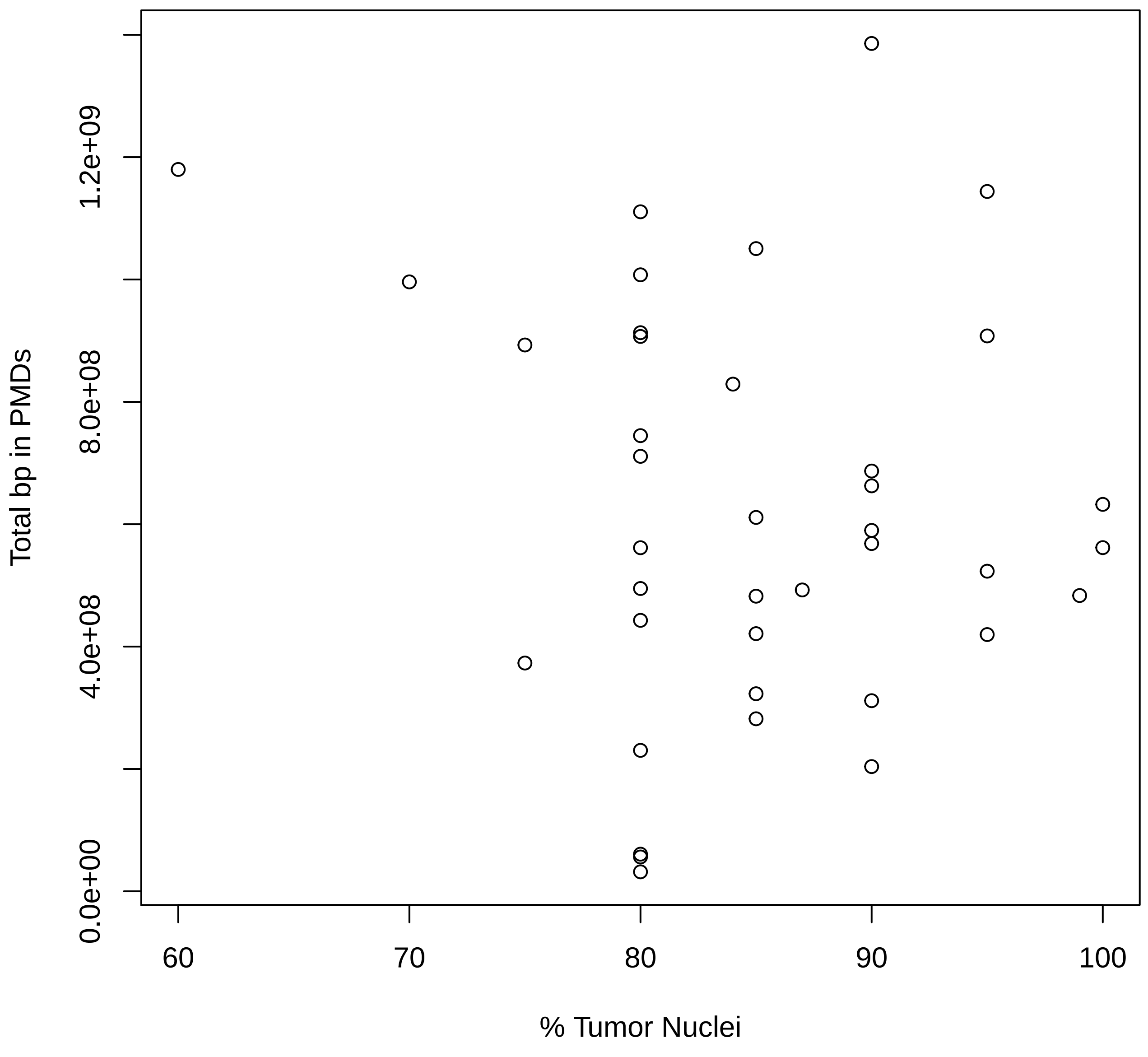

Figure S7. Scatterplot of TCGA-reported primary tumor purity against number of basepairs segmented into PMDs.

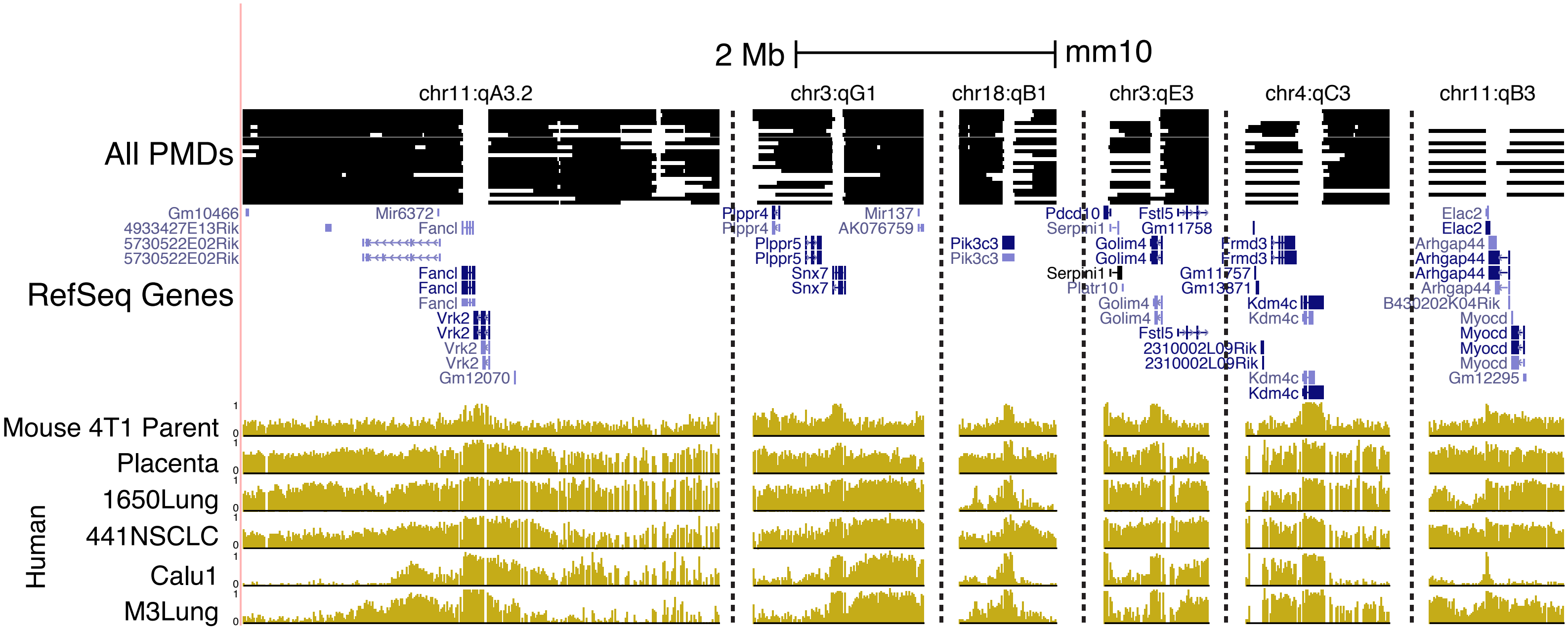

Figure S8. Top 6 most conserved mouse escapee genes and their methylation state in homologous regions of human.

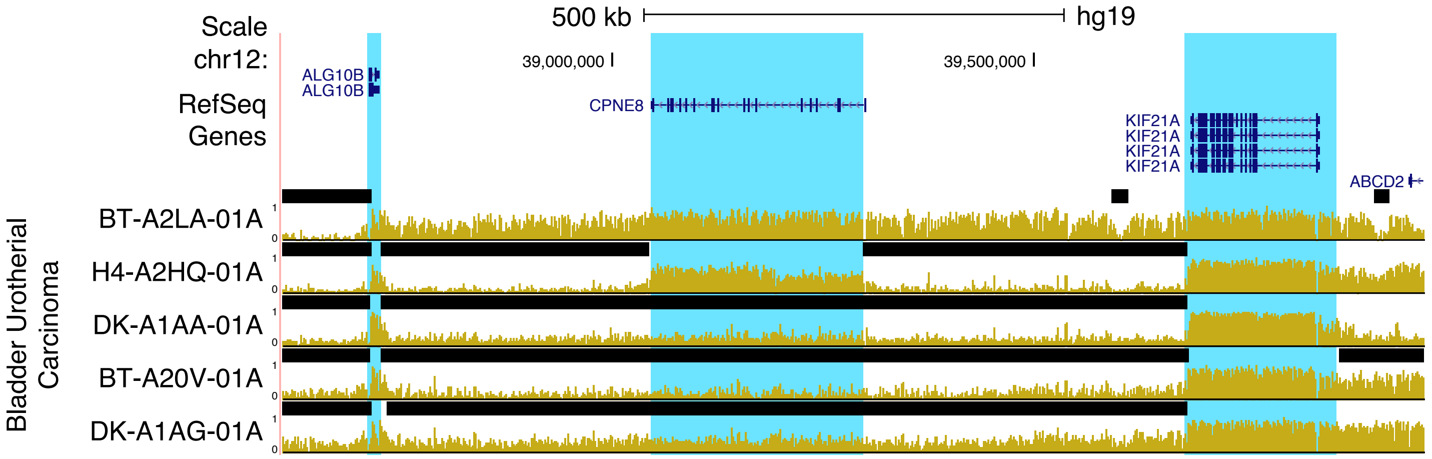

Figure S9. Adjacent escapee genes showing differential escapee state in TCGA bladder cancer samples.
